# Challenges and Strategies in the Industrial Application of *Dendrobium officinale*

**DOI:** 10.3390/plants13212961

**Published:** 2024-10-23

**Authors:** Hexigeduleng Bao, Hainan Bao, Yu Wang, Feijuan Wang, Qiong Jiang, Xiaoqi He, Hua Li, Yanfei Ding, Cheng Zhu

**Affiliations:** 1 Key Laboratory of Specialty Agri-Product Quality and Hazard Controlling Technology of Zhejiang Province, College of Life Science, China Jiliang University, Hangzhou 310018, China; 13848880640@163.com (H.B.); wangyu90203@163.com (Y.W.); wfj0311@cjlu.edu.cn (F.W.); qjiang@cjlu.edu.cn (Q.J.); dingyanfei@cjlu.edu.cn (Y.D.); pzhch@cjlu.edu.cn (C.Z.); 2 College of Engineering, Nanjing Agricultural University, Nanjing 210031, China; lihua@njau.edu.cn; 3 Ningbo Industrial Internet Institute Co., Ltd., Ningbo 315000, China; hexiaoqi@niii.com

**Keywords:** *Dendrobium officinale*, industrial development, ex vitro rapid propagation, pharmacological properties, germplasm resources

## Abstract

*Dendrobium officinale* Kimura & Migo (*D. officinale*) is a well-recognized traditional Chinese medicinal herb that is both medicinal and edible. Contemporary pharmacological studies have revealed that *D. officinale* contains abundant bioactive compounds, including polysaccharides, flavonoids, alkaloids, and dendrobine, exhibiting diverse pharmacological properties such as antioxidant, anti-inflammatory, and immunomodulatory effects. However, the industrial application of *D. officinale* faces many problems, such as the scarcity of wild resources, low natural reproduction rate, and slow growth rate as well as the lack of relevant industrial standards. Nevertheless, substantial advancements, including the exploitation of artificial propagation techniques and breeding of new varieties, have been achieved in recent years. These developments have effectively addressed the challenges associated with its low natural reproduction rate and the scarcity of wild resources. This review summarizes the progress in the industrial development, seedling cultivation, and pharmacological exploration of *D. officinale* in recent years. Furthermore, it analyzes current research inadequacies and offers strategic solutions to enhance its application in healthcare and medicine.

## 1. Background

*Dendrobium officinale* Kimura & Migo (*D. officinale*) is a traditional Chinese precious herb and food, with the reputation of being the “first among the nine immortal herbs of China” [1]. It contains various bioactive substances, including polysaccharides, flavonoids, alkaloids, dendrobium phenol, amino acids, phenanthrene, and benzyl compounds, which show diverse pharmacological properties such as antioxidation [2], treatment of osteoporosis [3], anti-inflammation [4], anti-aging [5], hypoglycemic [6], liver protection [7], and other effects. However, the industrial application of *D. officinale* faces many problems, such as the scarcity of wild resources, low natural reproduction rate, and slow growth rate. Recently, artificial fast propagation technologies have been developed and been widely used to achieve the sustainable utilization of *D. officinale* resources [8]. The expanding market for *D. officinale*, driven by its incorporation into pharmaceuticals, health supplements, foods, and cosmetics [9], not only capitalizes on the potential of traditional Chinese medicinal resources, but also addresses a market gap to facilitate the sustained growth of the *D. officinale* industry.

**Figure 1:**
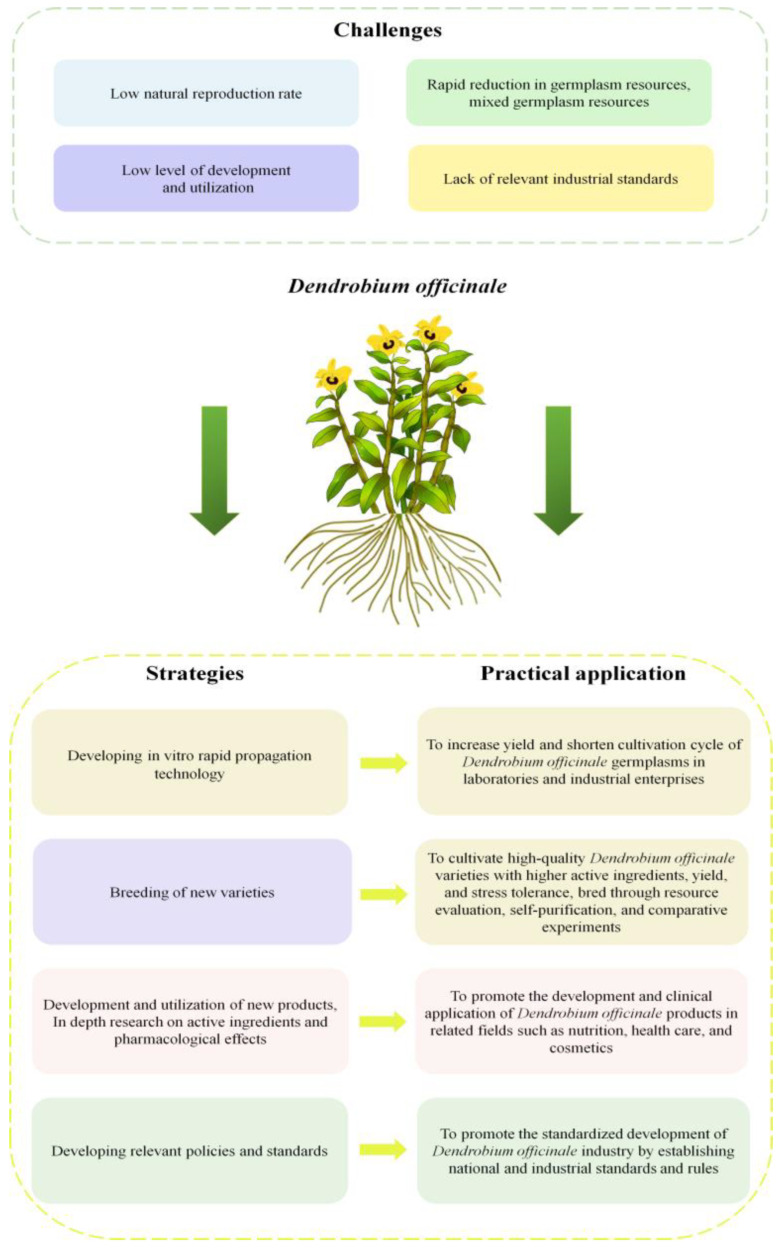
Challenges and strategies in the industrial application of *Dendrobium officinale*.

Currently, no national standards associated with the *D. officinale* industry have been issued in China. This situation has led to a predominant focus on yield at the expense of quality and the common malpractice of overusing pesticides, fertilizers, and growth-modifying chemicals. To broaden the applications of *D. officinale* and its derivatives, enhance their quality and safety, and minimize pesticide residues, localized standards have been issued in some provinces of China, such as Zhejiang, Yunnan, Guizhou, and Guangxi. These initiatives aim to advance the standardization and development of the *D. officinale* industry nationwide. Therefore, this review comprehensively summarizes the current state of the *D. officinale* industry, including development challenges, advancements in artificial propagation techniques, and research on pharmacological applications. It proposes targeted strategies such as strengthening the conservation of *D. officinale* germplasm resources, innovating pharmaceutical development and clinical usage, and establishing relevant national guidelines. These strategies are aimed at exploring effective approaches for accelerating the growth of the *D. officinale* industry, enhancing its societal and economic contributions, and facilitating the rapid expansion of *D. officinale*-based clinical treatments, as well as its broader applications in the healthcare services domain (Figure 1).

## 2. Current State of the *D. officinale* Industry

### 2.1. Progress in Pharmacological Activity Research on D. officinale

**Figure 2:**
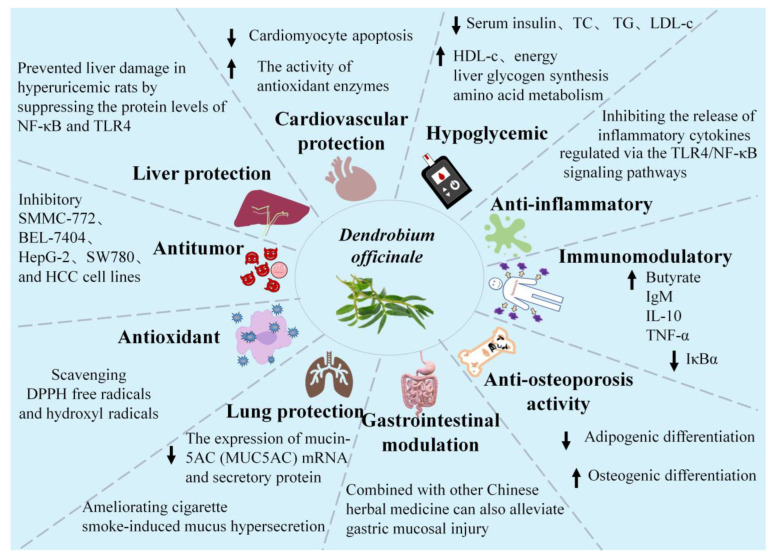
Pharmacological activities of *D. officinale*.

Existing research has successfully identified no fewer than 190 compounds from *D. officinale*, primarily including polysaccharides, phenanthrenes, bibenzyls, phenols, amino acids, and alkaloids [10]. The extensive diversity of these compounds underscores the broad spectrum of biological functionalities exhibited by *D. officinale*, including but not limited to cardiovascular protection, tumor inhibition, glycemic control, gastrointestinal health regulation, inflammation mitigation, immune system modulation, hepatoprotection, lung protection, antioxidation, and antiosteoporotic effects. The pharmacological activities of *D. officinale* are illustrated in Figure 2.

#### 2.1.1. Cardiovascular Protection

Cardiovascular diseases are a predominant cause of morbidity and mortality worldwide, primarily attributed to inflammation [11] and oxidative stress [12]. DOPs have been demonstrated to diminish malondialdehyde (MDA) activities while elevating superoxide dismutase (SOD) levels, thereby restricting intracellular reactive oxygen species (ROS) production and mitigating oxidative stress and apoptosis in H_2_O_2_-challenged H9c2 cells [13]. Subsequent research [14] has corroborated these findings, elucidating that DOPs protect H9c2 cardiomyocytes from oxidative stress by bolstering the antioxidant enzyme system, suppressing ROS accumulation, and modulating pro-apoptotic proteins. The anti-apoptotic effect of DOPs is associated with the regulation of the PI3K/Akt and MAPK signaling pathways. In another study, Yue et al. [15] isolated a novel sulfated polysaccharide from the stems of *D. officinale*, which exerted pronounced anti-angiogenic effects by notably hindering the migration of human microvascular endothelial cells (HMEC-1), even at low concentrations.

#### 2.1.2. Antitumor Effects

Extensive research has substantiated the potential of *D. officinale* in the prevention and treatment of tumors, which arise from a highly complex process. Its antitumor capabilities primarily involve bolstering immune response modulation and activating the expression of tumor suppressor genes [16]. Sun et al. [17] elucidated that DOPs stimulated the proliferative potential of spleen cells, the activities of natural killer cells and cytotoxic T lymphocytes, and the secretion of interferon-gamma (IFN-γ) and interleukin (IL)-10 by spleen cells in tumor-bearing mice. These findings posit DOPs as promising antitumor agents with prominent immunomodulatory properties. Further research [18] has indicated that DOPs can suppress the proliferation of the colon cancer cell line CT26 by increasing ROS generation, decreasing matrix metalloproteinase (MMP) levels, and activating the AMPK/mTOR autophagy signaling pathway. Another study [19] established a rat model of precancerous lesions of gastric cancer (PLGC) by administering *N*-methyl-*N*′-nitro-*N*-nitrosoguanidine (MNNG, 150 μg/mL) in the drinking water of male rats for 7 months. This regimen was supplemented with weekly applications of a 10% NaCl solution (0.1 mL) for the initial 20 weeks. The outcomes demonstrated that DOPs effectively inhibited MNNG-induced PLGC in rats, with the action mechanism linked to the Wnt/β-catenin pathway. Additionally, a notable elevation in betaine levels in endogenous metabolic products was observed following DOP treatment. Presently, a range of in vivo and in vitro evidence supports the antitumor properties of DOPs, underscoring the necessity for well-designed clinical trials to further assess their antitumor capabilities.

#### 2.1.3. Hypoglycemic Effects

Diabetes mellitus is a chronic metabolic disease, with an increasing incidence observed among the younger demographic, largely attributed to modern lifestyle factors such as obesity, sedentary behavior, smoking, poor dietary habits, a lack of physical activity, and adverse emotional states. Existing research suggests that improving cardiovascular health metrics could potentially reduce the risk of developing diabetes [20]. Moreover, diabetes can lead to complications such as nephropathy, cardiovascular and cerebrovascular diseases, and retinopathy. Emerging evidence supports the efficacy of herbal medicine in the management of diabetes and its associated sequelae [21,22]. In recent years, *D. officinale* has been extensively studied. Research has substantiated that administering low, medium, and high doses (75, 150, and 300 mg/kg) of *D. officinale* to streptozotocin/high-fat diet (STZ/HFD)-induced diabetic mice for 12 weeks alleviates lipid profiles by raising high-density lipoprotein cholesterol (HDL-c) levels and decreasing serum insulin, total cholesterol (TC), triglycerides (TG), and low-density lipoprotein cholesterol (LDL-c) levels [23]. Further investigations have validated the hypoglycemic potential of DOPs [24], highlighting their capacity to lower fasting glucose levels, reduce lipid concentrations, alleviate immune-related stress, and rectify metabolic imbalances associated with lipids, bile acids, and amino acids. Additionally, Liu et al. [25] elucidated the prophylactic benefits of DOPs against type 2 diabetes in murine models induced by a high-fat/high-sugar diet and STZ. This condition was attributed to their ability to modulate gut microbiota, reduce inflammatory responses, and enhance insulin sensitivity. Accordingly, forthcoming studies should prioritize the investigation of DOPs for the development and production of functional products or dietary supplements for diabetes prevention.

#### 2.1.4. Modulation of Gastrointestinal Function

*D. officinale*, together with its derived extracts, is extensively utilized in the treatment of gastrointestinal diseases. For instance, a study established a mouse model to simulate stomach yin deficiency [26] and evaluated symptomatic indicators (mouse body weight, food intake, water consumption, fecal water content, fecal pellet number, and foot temperature). The study revealed that the ultrafine powder of *D. officinale* alleviated these symptom indicators and mitigated gastric mucosal injuries. Furthermore, Ke et al. [27] demonstrated that *D. officinale* leaf polysaccharides (LDOP-1) effectively mitigated ethanol-induced gastric mucosal damage, a prevalent gastrointestinal condition, both in vivo and in vitro. This mitigation was achieved by reducing ROS accumulation and increasing SOD, total antioxidant capacity (T-AOC), and heme oxygenase-1 (HO-1) levels, suggesting that LDOP-1 might confer gastroprotection through the AMPK/mTOR signaling pathway. Additionally, fermentation could enhance the chemical composition of *D. officinale* polyphenols. Polyphenols in fermented *D. officinale* fluid improve intestinal health by enhancing immune activity in the gut, thereby altering the gut microbiota and metabolites [28].

#### 2.1.5. Anti-Inflammatory Properties

*D. officinale* exhibits remarkable anti-inflammatory activity. For instance, research into DOPs and their essential structural domain (EDOP) [29] has demonstrated their efficacy in protecting against colitis induced by dextran sulfate sodium in murine models. The findings indicate that both DOP and EDOP can mitigate pertinent symptoms by inhibiting serum levels and the mRNA expression of the pro-inflammatory cytokines tumor necrosis factor-α (TNF-α), IL-6, and IL-1β, elevating short-chain fatty acid levels, activating G-protein-coupled receptors, and modulating the gut microbiota. Another investigation by Liang et al. [30] revealed that DOPs ameliorated learning and memory impairments in mice, induced by ovariectomy (OVX) and D-galactose (D-GAL) through the upregulation of the Nrf2/HO-1 pathway. This mechanism prevented hippocampal neuronal cell damage caused by OVX and D-GAL and markedly alleviated neuroinflammation. While studies on its anti-inflammatory effects are still progressing and necessitate further in-depth in vivo studies and clinical trials, the development of anti-inflammatory drugs derived from *D. officinale* represents a promising avenue of research.

In addition to the previously outlined pharmacological properties, *D. officinale* has been validated to offer various therapeutic benefits, including antioxidation [2], hepatoprotection [31], lung protection [32], and antiosteoporosis actions [33]. The stem of *D. officinale* has consistently been a primary focus of research worldwide. Recent studies have established that the leaves of *D. officinale* exhibit similar chemical compositions [34] and pharmacological properties [27] to those of the stem. Lv et al. highlighted that HFD-induced mice displayed a notable reduction in blood glucose levels and an improvement in glucose tolerance following the administration of an aqueous extract from *D. officinale* leaves (EDL), indicating that the glucose-reducing effect of EDL might be associated with the activation of insulin signaling pathways [35]. Nevertheless, the exploration of the pharmacological potential of *D. officinale* leaves has been limited in recent years. Thus, delving into the function of *D. officinale* leaves and developing new products primarily composed of *D. officinale* leaves carry significant implications for the exploitation of new resources from *D. officinale*.

### 2.2. Industry Scale and Challenges in Development

*D. officinale* finds extensive application in the pharmaceutical, dietary supplement, and food industries, with a continuously growing demand observed in the market [36]. The industrial development of *D. officinale* in the southern regions of China, including Guangxi, Yunnan, Zhejiang, Fujian, Guizhou, and Jiangxi, has progressed rapidly, establishing a comprehensive and sizable industrial chain ranging from raw material cultivation to the manufacturing of healthcare products. In recent years, a favorable market trend for *D. officinale* has been observed, with a noticeable rise in the prices of seedlings in bottles and fresh products [37]. This trend has spurred the establishment of tissue culture factories and cultivation bases for *D. officinale* across various locations. Ongoing research into *D. officinale* has resulted in diversification of the types, dosage forms, and functional effects on the market. The industrialization of *D. officinale* is conducive to the development of deep-processed products and novel pharmaceuticals, thereby fostering a beneficial cycle of growth. Detailed information on the current development of medicinal products, health supplements, and cosmetics derived from *D. officinale* is provided in Table 1.

The *D. officinale* industry in China has undergone rapid development in recent years, leading to the establishment of an extensive industrial network [56]. Nevertheless, healthy and sustainable growth is hindered by several constraints, including the mixing of germplasm resources, the impact of counterfeit products, and unclear mechanisms behind product efficacy [57]. The exclusive dependence on differences in agronomic traits or singular technical cluster analysis proves insufficient for accurately distinguishing *D. officinale* sources. Recent advancements in cross-breeding, induced mutation breeding, genetic engineering, and molecular markers have opened avenues for genetic enhancements in *D. officinale*, potentially accelerating the plant’s development speed [58] and facilitating the rapid identification and authentication of its germplasm [59]. The development of *D. officinale*-based products is diverse and often formulated in conjunction with other traditional Chinese medicines to create health supplements. This practice complicates the differentiation of the listed polysaccharides and other components specific to *D. officinale*. Consequently, this issue impacts the expansion of marketing and the broader commercial reach of *D. officinale* products.

### 2.3. Current State and Application of D. officinale Seedling and Cultivation Techniques

#### 2.3.1. Progress in *D. officinale* Seedling Cultivation

Under natural conditions, the germination rate of *D. officinale* seeds is notably low, rendering large-scale sowing impractical. In response to growing market demands both domestically and internationally, rapid seedling propagation techniques have markedly advanced [60], leading to a notable reduction in the duration of seedling cultivation while maintaining seedling quality. This progress has further propelled the industrialization process of *D. officinale*. Currently, the primary methods of seedling cultivation for *D. officinale* include cutting propagation and tissue culture. The adept application of these two seedling cultivation methods has resolved the challenges of breeding *D. officinale* seedlings and achieved sustainable, low-carbon seedling production.

The cutting propagation of *D. officinale* is recognized for its cost-effectiveness, simplicity, and preservation of the parent plant’s characteristics. Notably, soaking cuttings in a solution comprising thidiazuron (TDZ) (200 mg/L), naphthaleneacetic acid (NAA) (100 mg/L), and indole-3-butyric acid (IBA) (200 g/L), and utilizing sphagnum moss as the cutting substrate, yields a budding rate of 80.0–85.5% [61]. Optimal results in bud differentiation are attained by selecting material from the upper portion of the pseudobulb for cuttings. Furthermore, Zhao et al. [62] innovated a tailored cutting propagation technique for the Guangdong region incorporating the soaking of *D. officinale* cuttings in a pharmacological solution within a dark environment, thereby successfully increasing the sprouting rate to above 60%.

The traditional approach to propagating *D. officinale* through cuttings faces several issues, including inconsistent seedling size, weak growth potential, and low quantities of seedlings produced in a single batch. These challenges hinder the possibility of standardized, large-scale cultivation [63]. Hence, the application of ex vitro tissue culture techniques has been proposed to enhance the success rate of developing mature plants and ultimately accelerate the propagation speed of *D. officinale* [64,65]. Recent research has focused on critical aspects of *D. officinale* tissue culture, including the careful selection of explants, the refinement of culture media, and the exploration of various factors influencing the success of tissue cultures.

In the field of tissue culture, seeds, protocorms, and organ explants are employed in studies focusing on medium optimization, the application of exogenous hormones and additives, and the influence of environmental factors such as temperature, light, and pH on the development of tissue-cultured seedlings (Table 2). Notably, seed explants have been extensively studied due to their notably high germination and proliferation differentiation rates. Qiu et al. [66] revealed that seeds germinated more efficiently and rapidly in half-strength Murashige and Skoog (1/2 MS) medium compared to full-strength Murashige and Skoog (MS) medium. Tang et al. [67] successfully induced bud formation using vigorous stem segments with nodes from *D. officinale* as explants, establishing an efficient system for asexual propagation. Through orthogonal experimentation, Xie et al. [68] determined that a cultivation medium comprising MS supplemented with 2,4-dichlorophenoxyacetic acid (2,4-D, 0.2 mg/L), NAA (0.8 mg/L), and kinetin (0.4 mg/L) was the most favorable for the proliferation of *D. officinale* protocorms. Ma et al. [69] evaluated the impact of various media on seed germination and subsequent robust seedling development in *D. officinale*. Their experiments optimized a rapid propagation system by incorporating various plant growth regulators and additives. They found that a medium consisting of 1/2 MS enriched with NAA (0.4 mg/L), 6-benzylaminopurine (6-BA, 0.1 mg/L), sucrose (20 g/L), agar (6 g/L), banana puree (80 g/L), and activated charcoal (0.5 g/L) yielded the best results for robust rooting and seedling growth. Temperature, light intensity, and the pH of the culture medium are critical factors affecting the quality of tissue-cultured seedlings. The research conducted by Li et al. [70] highlighted that a light intensity of 75 μmol/(m^2^·s) prominently fostered stem girth, weight, rooting efficiency, leaf soil plant analysis development (SPAD) values, and sucrose synthase activity in ex vitro *D. officinale* seedlings. Moreover, Liu et al. [71], through controlling humidity, light intensity, and photoperiod, explored the impact of temperature on the quality of *D. officinale* tissue-cultured seedlings. Their findings unveiled the optimal cultivation temperature to be 25 °C. Chen et al.’s [72] investigation into the effect of pH under consistent medium, light, and temperature conditions demonstrated that a pH of 5.4 facilitated the optimal growth and development of *D. officinale* tissue-cultured seedlings.

#### 2.3.2. Current State of *D. officinale* Cultivation Techniques

To conserve wild *D. officinale* resources while keeping up with the growing market demands, China has notably advanced the *D. officinale* cultivation industry in recent years [73]. By innovating cultivation methods, the supply-and-demand imbalance has been effectively alleviated. The main cultivation modes include greenhouse cultivation and semi-wild cultivation. Additionally, “Technical Procedures for *Dendrobium officinale* Cultivation” have been formulated to provide guidance and standardization for these practices.

Greenhouse cultivation optimizes the environmental conditions necessary for *D. officinale*, thereby enhancing its survival rate and substantially boosting yield. The survival rate of *D. officinale* seedlings is critically influenced by the choice of cultivation substrate, which requires excellent aeration, water permeability, and robust nutrient and moisture retention. The substrates currently employed for *D. officinale* include various materials, such as biowaste bacterial dregs, biochar, bark, sphagnum moss, vermiculite, perlite, coconut coir, peanut shells, and charcoal [74]. Qin et al. [75] investigated the effects of diverse substrates on the yield and quality of *D. officinale* and revealed that using pine bark as a substrate not only increased the survival rate by 6.76%, but also enhanced plant height, stem girth, and dry weight by 4.57%, 4.27%, and 9.72%, respectively, with an 11.11% increase in protein content, proving its effectiveness for northern cultivation areas. Furthermore, Tan et al. [76] assessed the influence of oyster mushroom dregs, pine scales, and a composite substrate on *D. officinale* quality and polysaccharide content. Their findings pinpointed the composite substrate (V_mushroom dregs_:V_pine scales_:V_gravel_ = 6:2:2) as superior in quality, polysaccharide content, and water retention. Additionally, Zhu et al. [77] identified the optimal growth conditions for seedlings with a substrate mix of pine bark to brick slag at a 7:3 ratio, 75% air humidity, and a substrate moisture range of 30–35%. Temperature, humidity, and light are crucial factors affecting the growth of *D. officinale* seedlings. Zhang et al.’s research [78] indicated that the optimal net photosynthesis rate for *D. officinale* seedlings occurs at 20~25 °C, humidity over 80%, and a light intensity of 240 μmol·m^−2^·s^−1^. Reasonable fertilization strategies are pivotal for growth enhancement, with current studies focusing on controlled-release, water-soluble, and organic fertilizers. Kang et al.’s study [79] on suitable greenhouse fertilization regimes to enhance growth and yield demonstrated that a combination of controlled-release and organic liquid fertilizers can improve survival and growth rates while saving costs and labor, thereby potentially facilitating the widespread adoption of this approach. Qian et al. [80] conducted experiments involving 1-year-old and 2-year-old potted seedlings of *D. officinale* and found that the utilization of slow-release fertilizers evidently boosted polysaccharide, extract, and total amino acid contents.

The intensification of planting densities and the extensive use of fertilizers, plant hormones, and pesticides in greenhouse cultivation have raised considerable concerns regarding the accumulation of toxic and harmful substance residues. This challenge has prompted the development of a semi-wild cultivation model. Understory biomimetic cultivation and lithophytic epiphytic cultivation are two primary methods of wild-simulated cultivation for *D. officinale*. Understory biomimetic cultivation should consider factors such as environmental stability, humidity, canopy density, bark texture, and natural bark desquamation. In the climatic context of the Emei Mountains, Gu et al. [81] utilized plum trees, a local economic fruit tree, as host trees for the epiphytic cultivation of *D. officinale*. Their findings highlighted the vitality of *D. officinale*, contributing to both product safety and economic efficiency. Comparative studies by Liang et al. [82] explored four different cultivation methods: understory epiphytic planting (suspended branch planting, tree body planting), understory seedbed planting, and brick–stone planting, revealing that epiphytic planting methods yield higher propagation and weight increase coefficients for *D. officinale*. When *Artocarpus heterophylla*, longan, mango, lychee, and Masson pine trees were used as host trees, the first three tree species exhibited higher survival rates, indicating that evergreen broad-leaved trees are conducive to epiphytic planting. Luo et al. [83] investigated the wild-simulated cultivation techniques of *D. officinale* in the karst landscapes of Guizhou and suggested optimal growth rates and cultivation outcomes in open, well-ventilated locations with sufficient sunlight, particularly in the middle to upper parts of mountains with 30–50% canopy coverage.

The environmental conditions in which a plant grows primarily affect the concentration of its constituents and the levels of specialized metabolites [84,85]. Notably, the yield and active ingredients of *D. officinale* vary widely across different cultivation modes. Yu et al. [86] demonstrated that, following oxidative degradation, polysaccharides from *D. officinale* grown in semi-wild conditions exhibited markedly enhanced antitumor properties compared to those cultivated in greenhouses. These degraded polysaccharides induced apoptosis in HeLa cells through the p38/MAPK signaling pathway. A further investigation by Yang et al. [87] analyzed the metabolites of *D. officinale* under various cultivation methods (living tree epiphytic cultivation, lithophytic epiphytic cultivation, and greenhouse cultivation). Their data delineated that the content of *D. officinale* polysaccharides (DOPs) from greenhouse and lithophytic epiphytic cultivation surpassed that from living tree epiphytic cultivation. Moreover, *D. officinale* cultivated lithophytically exhibited the most robust antioxidant capabilities compared to its less effective greenhouse-grown counterparts. These findings underscore the profound impact of the growth environment on the chemical composition of *D. officinale*. While semi-wild cultivation offers numerous benefits, including lower planting density, increased resistance to pests and diseases, reduced chemical residue, and a higher concentration of active components, it is hindered by higher labor costs and diminished yields. Future research aimed at broader adoption should prioritize strategies to enhance yield.

## 3. Strategies for Industrialization of *D. officinale*

### 3.1. Enhancement of the Conservation of D. officinale Germplasm Resources

#### 3.1.1. Advancements in Ex Vitro Rapid Propagation Techniques for *D. officinale*

Since the late 20th century, China has achieved significant advancements in rapid propagation techniques for *D. officinale* [88]. Seeds, extensively examined as explants in these methods, offer distinct advantages, including a notably lower infection rate and greater uniformity of the resulting seedlings compared to the induction of stem segments [89]. This method is acknowledged for its simplicity, potent proliferation, and differentiation capabilities, meeting the demands of industrial-scale seedling production for *D. officinale*. Nevertheless, the variability in seed characteristics poses challenges in ensuring the uniformity and quality of the seedlings, with a propensity for variations in subsequent generations. Conversely, utilizing stem segments as explants has the advantage of shortening the culture period and preserving the characteristics of the parent plant, albeit at the expense of lower productivity. Therefore, exploring new induction methods for producing high-quality *D. officinale* stem segments, reducing the infection rate during the cultivation period, and implementing methods to enhance yield represent emerging trends in the development of the *D. officinale* industry (Figure 1).

#### 3.1.2. Selection and Breeding of Superior *D. officinale* Varieties

Germplasm, cultivation modes, and environmental conditions are crucial factors influencing the production of high-quality *D. officinale*. Among these, germplasm resources serve as the foundation for identifying and breeding superior varieties. *D. officinale* resources in China are predominantly concentrated in provinces such as Yunnan, Zhejiang, Guangxi, Guizhou, Anhui, and Fujian. However, the marked decline in wild germplasm resources and the amalgamation of genetic materials in recent years have posed challenges to the breeding of superior *D. officinale* varieties. In modern research, molecular biology and bioinformatics methods have been extensively employed to identify and evaluate *D. officinale* germplasm resources [90,91]. There is an urgent need to expand the application of modern molecular biology methods to provide novel avenues for comprehensive investigation into the growth, identification, metabolic regulation, and utilization of *D. officinale*. Selective breeding and cross-breeding, recognized as the most effective genetic breeding methods in *D. officinale* cultivation, confer advantages in preserving desirable attributes, enhancing yield and quality, innovating germplasm resources, and fortifying stress resistance. Basic information on *D. officinale* varieties, including clearly delineated sources and detailed characteristic descriptions, is summarized in Table 3. It is of substantial importance to enhance the selection and breeding of superior *D. officinale* varieties to guide the research, production, and cultivation of high-quality *D. officinale* (Figure 1).

#### 3.1.3. Progress in the Enhancement of the Yield and Quality of *D. officinale*

The indiscriminate introduction and mixing of germplasm in the cultivation of *D. officinale* have led to significant discrepancies in both quality and yield, hampering the systematic development of the *D. officinale* industry. To address yield improvements, forthcoming studies should prioritize enhancing the survival rate and growth speed of *D. officinale* tissue culture seedlings upon transplantation. Moreover, artificial cultivation techniques play a crucial role in influencing the yield and quality of *D. officinale*. Emphasis should be placed on cultivation modes, environmental conditions, reducing chemical fertilizer and pesticide usage, and disease and pest management to establish an economically and environmentally sustainable, productive, and high-quality cultivation approach for *D. officinale*. The strategic utilization of endophytic fungi has been recognized as a potent means to markedly enhance both the yield and quality of *D. officinale*, rendering the exploration of endophytic fungi’s role in plant growth a pivotal area of focus.

In light of escalating market demand driven by socio-economic growth, there is a compelling imperative to bolster the production of *D. officinale* through the expansion of its cultivation areas. The need to expand cultivation zones and enhance the quality of raw materials has led to a gradual northward shift in the cultivation regions of *D. officinale*. Furthermore, this imperative highlights the critical importance of developing frost-resistant *D. officinale* varieties to achieve the goals of increased production and expanded cultivation areas.

### 3.2. Advancements in Pharmaceutical Development and Clinical Integration of D. officinale

Currently, the exploration of the biological activities of *D. officinale* has predominantly focused on in vitro and animal models, with a notable scarcity of clinical studies assessing health implications in humans. This gap underscores the imperative to expound upon its potential in clinical drug formulations and health supplements. Detailed and large-scale clinical trials are needed to evaluate the bioactivity of *D. officinale* and its therapeutic effects on various diseases, thereby affirming the safety, reliability, and efficacy of its pharmacological activities. Additionally, intensified research on the pharmacokinetics of *D. officinale* is warranted to explore its absorption and metabolism within the body, determine optimal dosages, and further advance the drug development and clinical application of *D. officinale* (Figure 1).

### 3.3. Establishment of Relevant Policies and Standards

With the increasing emphasis on health management, the “food-medicine homology” industry is poised for broad development prospects [104]. As a tangible embodiment of this concept, *D. officinale* has been gradually integrated into the regulatory framework governing food substances in China. On 6 January 2020, the National Health Commission issued a directive to pilot the management of substances traditionally recognized for their roles as both food and medicinal materials. This directive encompassed *Codonopsis pilosula* and eight other substances (including *D. officinale*), with plans to potentially incorporate these substances into the regulatory framework governing food and medicinal substances based on the outcomes of the pilot initiatives. Pilot projects, considering *D. officinale* as both a food and a medicinal herb, are currently underway in provinces such as Shandong, Yunnan, Zhejiang, and Jiangxi.

Despite the adaptation and implementation of local standards in key *D. officinale*-producing regions, the establishment of a national production standard is still pending. Zhejiang Province has developed provincial food safety standards for “Dried *Dendrobium officinale* Leaves” and “Dried *Dendrobium officinale* Flowers”, thereby stimulating the advancement of the food and medicinal substance industry. The formulation of the International Organization for Standardization (ISO) for “Traditional Chinese Medicine *Dendrobium officinale*” has significantly propelled the integration of Zhejiang’s traditional Chinese medicine quality norms with international standards. Future endeavors should prioritize expediting the establishment of national standards for *D. officinale* production to foster the standardized development of the *D. officinale* industry nationwide (Figure 1).

## 4. Conclusions

*D. officinale*, a product with both medicinal and dietary origins, plays a pronounced role in promoting health and wellness. Presently, an expanding body of research highlights the importance of structure-based drug design in the development of novel pharmaceuticals. Further investigations are warranted into the structural modifications, structure–activity relationships, activation mechanisms, and biosynthesis of *D. officinale* compounds. Moreover, exploration should extend to the identification and isolation of novel compounds within *D. officinale*. In addition to the predominant focus on its stems, recent studies such as Chen et al. [105] provide insights into the reproductive development of *D. officinale*, particularly in the formation of integuments and megasporogenesis. These findings could have significant implications for understanding the cultivation and genetic optimization of the species, suggesting that more attention should be given to other parts of the plant, such as its flowers, leaves, and roots, to fully exploit its therapeutic and practical benefits. In addition, the quality of *D. officinale* is susceptible to various influences during cultivation, storage, and processing, underscoring the necessity for robust national and industry-specific standards. These measures are pivotal for fostering standardization and growth within the *D. officinale* industry. Ultimately, the advancement of the *D. officinale* industry not only protects valuable genetic resources, but also offers broad prospects for product innovations in the domains of food and pharmaceuticals.

## Figures and Tables

**Table 1:** Development and application of *D. officinale* in pharmaceuticals, health foods, and cosmetics.

Product Name	Materials	Effect	Ref.
Teipi Fengdou granules	*D. officinale*, *Panax quinquefolius* L., glucose, etc.	Anti-inflammatory	[38]
*Dendrobium* compound granules	*D. officinale*, *Panax quinquefolius* L., etc.	Immune enhancement	[39]
*D. officinale Ganoderma lucidum* wine	*D. officinale*, *Ganoderma lucidum*, wine	Enhancement of immune function in mice	[40]
*D. officinale* vinegar	*D. officinale* pulp, rice saccharification liquid	Health protection	[41]
*D. officinale* eye-brightener pill	*D. officinale*, *Lycium chinense Miller*, Cassia seed, etc.	Nourishment of Yin and brightness of eyes	[42]
*Dendrobium candidum* health noodles	*D. officinale*, flour	Health preservation	[43]
Beverage	*D. officinale*	Health care function	[44]
Maca and *D. officinale* tablets	Maca, *D. officinale*	Development of immunity against disease	[45]
Essence	*D. officinale* extract	Antioxidant effect and inhibition of tyrosinase activity	[46]
Emulsion	*D. officinale*	Skin moisturization	[47]
Facial cleanser	*D. officinale* extract, *Angelica keiskei Koidz.* extract, *Verbena officinalis* L. extract, etc.	Gentle and non-irritating effect	[48]
Moisturizing toner	*D. officinale* flower, glycerin, ethanol, etc.	Skin moisturization	[49]
Spray	*D. officinale* extract, *L-carnosine*, betaine, etc.	Anti-aging properties and enhancement of facial firmness	[50]
Bath lotion	*D. officinale* extract, *Panax ginseng* extract, *Selaginella tamariscina* (*P. Beauv.*) *Spring* extract, etc.	Efficient sterilization and antibacterial activity	[51]
Hand cream	*D. officinale* extract, *Panax ginseng* extract, tocopherol, etc.	Inflammation reduction and skin soothing	[52]
Face cream	*D. officinale* extract, licorice extract, squalane, etc.	Anti-inflammatory repair and allergy relief with moisturizing benefits	[53]
Handmade soap	*D. officinale* extract, anthocyanidin, palm oil, etc.	Free radical clearance and skin hydration	[54]
Lip gloss	*D. officinale* concentrated juice, rose fruit oil, honey, etc.	Moisturization and antioxidative properties	[55]

**Table 2:** Current state of tissue culture research on *D. officinale*.

Research Direction	Materials and Influencing Factors	Ref.
Selection of explants	Seed	[66]
	Roots, buds, stems	[67,68]
Optimization of culture medium	Proliferation and differentiation medium	[68]
	Rooting and strong seedling culture medium	[69]
	seed germination medium	[69]
Other influencing factors	Exogenous hormones such as 6-BA, NAA, IBA, etc.	[69]
	External additives such as coconut milk, banana puree, etc.	[69]
	Illumination	[70]
	Temperature	[71]
	pH	[72]

**Table 3:** New varieties of *D. officinale*.

Variety	Phenotypic Characteristics	Quality Characteristics	Source	Ref.
Yuhu 4	SH: 30–50 cm; SD: 0.8–10 cm; LL: 5–8 cm; LW: 1.5–3.0 cm; ID: 2.0–3.5 cm; R: 3–6 cm	PC: >40%	FP: Domestication of wild resources from Yunnan; MP: Domestication of wild resources from Yandang Mountains	[92]
Taihu 1	SH: 15.2 cm; SD: 0.5 cm; LL: 4.3 cm; LW: 1.2 cm; ID: 1.7 cm	PC: 32.4% MC: 15.4%	FP: Domestication of wild resources from Taining; MP: Domestication of wild resources from Leqing	[93]
Fuhu 1	SH: 20.7 cm; SD: 0.52 cm; LL: 5.0 cm; LW: 1.6 cm; ID: 1.5 cm	PC: 51.8% MC: 29.3% R_M/G_: 2.5 EEC: 15.3% WC: 8.1%	Domestication of wild resources	[94]
Huihu 1	SH: 15–30 cm; SD: 0.8–1.5 cm; LL: 2–4.5 cm; LW: 0.8–2.0 cm; ID: 0.3–1.8 cm; R: 2–4 cm	PC: 38.1% EEC: 6.9%	Domestication of wild resources from Dabie Mountains	[95]
Huihu 2	SH: 15–35 cm; SD: 0.6–1.2 cm; LL: 2.2–4.8 cm; LW: 0.8–1.5 cm; ID: 0.6–2.5 cm; R: 2–4 cm	PC: 37.3% EEC: 6.8%	Domestication of wild resources from Dabie Mountains	[96]
Qinggu 1	SH: 29.7–36.7 cm; SD: 0.972–1.1 cm	PC: 47.6%	Domestication of wild resources	[97]
Yanchuixue 3	SH: 25.5 cm; SD: 0.6 cm; LL: 4.8 cm; LW: 1.5 cm	PC: 57.5% MC: 32.6% R_M/G_: 2.3	Hybrid of green-stem (Tiantai Mountains) and red-stem (Yandang Mountains) plants	[98]
Guihu 1	SD: 0.3–0.8 cm; LL: 2–7 cm; LW: 0.5–2.5 cm	PC: 35.9%	Domestication of wild resources	[99]
Xianhu 1	SH: 20–30 cm; SD: 0.5–1 cm LL: 4–7 cm; LW: 1–2 cm; R: 2–4 cm	PC: 42.7% MC:	Domestication of wild resources	[100]
Xianhu 2	SH: 30–50 cm; SD: 0.6–1 cm LL: 4–7 cm; LW: 1–2.5 cm; ID: 1.5–2.5 cm; R: 2–4 cm	PC: 39.6% MC: 23.2%	Domestication of wild resources	[101]
Xianhu 3	SH: 18–21 cm; SD: 0.2–0.6 cm; LL: 2–7 cm; LW: 0.8–2.2 cm; ID: 0.8–1.4 cm; R: 4–6 cm	PC: 33.41% MC: 21.7% EEC: 13.8% R_M/G_: 4.4	FP: No. 514 MP: Xianhu1	[102]
Guijinghu 1	SH: 18–50 cm; SD: 0.4–0.7 cm; LL: 5–6 cm; LW: 1–1.8 cm; ID: 3–6 cm	PC: 32.3% MC: 23.6% EEC: 3.3%	Selection of good individuals from wild seedlings (Nazuo Miao Nationality Township, Xilin County, Guangxi)	[103]
Guijinghu 2	SH: 35–45 cm; SD: 0.4–0.6 cm; LL: 5–6 cm; LW: 1–2 cm; ID: 1–6 cm	PC: 43.6% MC: 21% EEC: 3.3%	Selection of good individuals from wild seedlings (Nazuo Miao Nationality Township, Xilin County, Guangxi)	[103]

SH: stem height; SD: stem diameter; LL: leaf length; LW: leaf width; ID: internode distance; R: raceme; PC: polysaccharide content; MC: mannose content; EEC: ethanol-soluble extract content; R_M/G_: chromatographic peak area ratio of mannose to glucose; WC: water content; FP: female parent; MP: male parent.

## Data Availability

No new data were created or analyzed in this study. Data sharing is not applicable to this article.
